# The road to a sustainable energy system in the Guadeloupe archipelago: Challenges and opportunities

**DOI:** 10.1016/j.heliyon.2025.e41760

**Published:** 2025-01-14

**Authors:** Mostafa Barani, Konstantin Löffler, Luka Garibashvili, Pedro Crespo del Granado

**Affiliations:** a Department of Industrial Economics and Technology Management, NTNU, Trondheim, Norway; b Workgroup for Infrastructure Policy, TU Berlin, Berlin, Germany

**Keywords:** Guadeloupe, Power, heat and transportation sectors, CO_2_ emission reduction, Sustainable transition of energy systems, Energy system modeling

## Abstract

The Guadeloupe archipelago, situated in the eastern Caribbean Sea with a population of approximately 400,000 inhabitants, faces distinctive challenges in realizing a sustainable and resilient energy transition owing to its nature being a distributed island system. These unique challenges include the absence of energy interconnections, limited domestic energy resources, substantial dependence on fossil fuels, and considerable load variance. These factors necessitate specialized attention in the study and analysis of the energy system transition. In this study, the authors initially delineate storylines tailored to the specific requirements of Guadeloupe's energy system, with a primary focus on the reliance on fossil fuel imports. Two distinct energy transition roads, aiming for self-sufficiency and independence for Guadeloupe by 2040 and 2050, are established. Leveraging an advanced open-source investment and dispatch tool, the Global Energy System Model (GENeSYS-MOD), these storylines are implemented to ascertain the least-cost, sustainable, and resilient energy transition for the Guadeloupe archipelago. The ensuing results are comprehensively analyzed to offer insights into Guadeloupe's trajectory towards a sustainable energy system, comparing the proposed energy transition roads for achieving sustainability. The findings reveal that substantial investments in wind and photovoltaic power plants, coupled with electricity storage devices, are imperative for achieving energy independence in the future. Additionally, the availability of biomass emerges as a crucial factor facilitating a seamless transition across all sectors, encompassing power, heat, and transportation.

## Highlights

• Study addresses Guadeloupe's fossil fuel dependency and energy security risks.
• Proposes solutions for significant CO_2_ emission reductions and energy self-sufficiency.
• Investment in wind and PV critical for Guadeloupe's energy independence by 2050.
• Electricity sector is the first and most important to decarbonize.
• Current government targets are not sufficient to reach energy independence.

## 1 Introduction

### 1.1 Aim and scope

The current energy supply of the Guadeloupe archipelago is largely based on imported fossil energy carriers such as oil, petroleum-derivatives, and coal with a total fossil share of over 90% of total energy consumption in 2020 [1]. This heavy dependence on fossil fuels has led to several adverse impacts, including two significant challenges that require urgent attention:

• Firstly, the current energy supply has a substantial carbon footprint, which contributes significantly to the environmental degradation of the region.
• Secondly, Guadeloupe's heavy reliance on imported fossil fuels also poses a significant risk to the region's energy security. With almost all of its primary energy coming from imports, Guadeloupe is highly vulnerable to disruptions in its supply chain. Any disruptions, whether due to natural disasters, geopolitical tensions, or other factors, could have severe consequences for the region's economic growth and development.

The primary objective of this study is to quantitatively analyze and address the challenges faced by the Guadeloupe archipelago in achieving a sustainable and resilient energy transition. It is worth noting that the regional government of Guadeloupe has taken several steps to promote energy independence and transition away from fossil fuels. The most recent policy, the Energy Transition for Green Growth Act (LTECV), is a national act that mandates the creation of Guadeloupe's own Multi-Year Energy Plan (PPE) (Article 203) [2]. Following the adoption of the LTECV in 2015, the first iteration of the PPE, for the timeframe of 2016–2023, was adopted in 2017 (with more ambitious goals and targets compared to its predecessors) and has since been used as the primary framework for energy planning on the archipelago by the relevant authorities and organizations [3]. The primary objective of the PPE is to ensure the effective fulfillment of the requirements set out by the LTECV requirements, particularly relating to final energy consumption, such as achieving a share of 50% from renewable sources in the final energy consumption by 2020 and energy independence by 2030. The PPE also provided sectoral targets towards sustainable transport, the addition of new renewable capacities to the electricity system, and decreasing overall energy and electricity demand. The policy document is heavily focused on reinforcing energy independence and bolstering the utilization of domestic resources to curb fossil fuel consumption. It should be noted that despite overall improvements in renewable energy penetration and strengthened energy security, the PPE failed to achieve the set goals for 2020 (with a 7% share of renewables instead of 50%) and still relied heavily on imports for all energy needs (with a 92.81% import dependency in 2020). Given that the current PPE is nearing the end of its intended lifespan, local authorities and stakeholders are working on revising the policy and drafting a more achievable scenario for the development of the energy sector of the archipelago. In the face of this, additional energy modeling is imperative to maintain a realistic approach towards the needed energy transition and outline some of the main development opportunities and no-regret options for the system in the future.

While there have been several studies on the energy systems of one or several Caribbean islands, such as Shirley and Kammen [4], with a study on Grenada, Barbados, Jamaica and the Netherland Antilles, Harrison and Popke [5], with a study on Dominica, Jamaica, Grenada, St. Lucia, and St. Vincent, and the Grenadines, and Hoody et al. [6] with a paper on the island nation of Antigua and Barbuda or the entire Caribbean region as a whole [7], [8], [9], [10], [11], [12], there is a substantial lack of academic literature on the Guadeloupe archipelago itself. Out of the aforementioned sources, two did not consider Guadeloupe at all [7], [10], three of them grouped Guadeloupe into a larger cluster of regions [9], [11], [12], and Dominković et al. [8] did not specifically portray a case study for one distinct Caribbean island or nation, but instead “depicts a typical island nation in the Caribbean”. Also, most of the studies have only analyzed the electricity system without consideration of the other energy sectors and their interconnections. Only Kunkar [11] features a sector-coupled approach, but analyzes the Caribbean region as a whole and thus clusters Guadeloupe together with several other islands. Therefore, this paper contributes to existing literature by presenting a spatially detailed representation of the energy system in Guadeloupe, together with several transition pathways towards sustainable and renewable energy systems.

## 2 Modelling the energy landscape

### 2.1 Implementation and methodology

To compare several long-term development options for Guadeloupe, we formulated multiple scenarios towards 2050: a baseline energy transition scenario without any CO_2_ emission constraints, alongside a set of alternative energy transition roads designed to address the key challenges of import dependency and sustainability. These transition scenarios are principally shaped by two pivotal factors: 1) a constraint on imports, particularly fossil fuels, aimed at securing Guadeloupe's energy self-sufficiency and independence, and 2) a cap on CO_2_ emissions. Both of these limiting factors function in a similar fashion, curbing investments in conventional generation resources. Consequently, the more stringent constraint effectively curtails the utilization of these resources.

We then employed the open-source Global Energy System Model (GENeSYS-MOD) to determine the most cost-effective solution for each of the storylines. The energy supply of Guadeloupe currently relies on oil and coal-based generation resources, as well as some renewable energy sources, namely photovoltaic (PV), wind, biomass, geothermal, and hydro. The model employs the initial set of generation capacities and resource availability and matches them with the sectoral energy demands to determine the optimal generation capacities, storages, and energy mix that meet the targets established in each scenario. Each model run covers the period from 2018 to 2050.

### 2.2 GENeSYS-MOD: a cross-sectoral approach to long-term energy pathways

The Global Energy System Model (GENeSYS-MOD) is an open-source, cross-sectoral energy system model specialized in computing long-term energy pathways, mostly focusing on heavily decarbonized energy systems [13]. The modeling framework can be applied to a wide range of energy-related research questions on multiple regional levels, from the global scale or macro-regions down to municipalities, providing the least-cost pathways for the future energy system. The model has been applied to several regional and country-level case studies [14], [15], [16], [17], [18], and provides insights into the sectors of electricity, transport, buildings, and industry. To compute the cost-optimal transition pathway, GENeSYS-MOD uses a set of linear equations and detailed sectoral input data on technologies, resource potentials, and demand developments. The model then minimizes the costs over the entire modeled time period, calculating the needed investments into technologies, storages, transformation, and transmission to fulfill the final sectoral energy demands. GENeSYS-MOD is available on GitLab, with many public data sets being published on Zenodo [19], [20].

**Figure 1:**
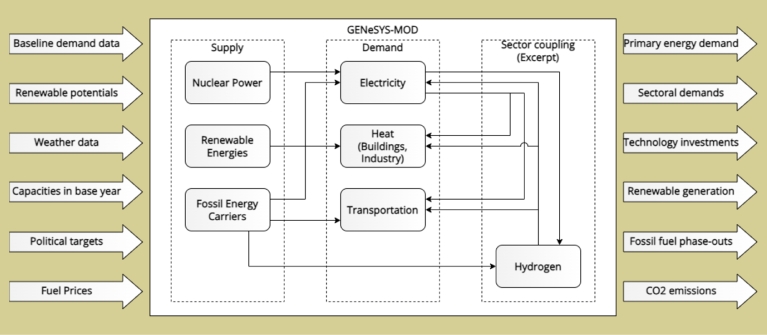
Structure of the GENeSYS-MOD model.

The model relies on several important input parameters for effective implementation, as shown in Fig. 1. The main initial data evaluated for this include:

• Baseline demand data – existing demand for energy (for this particular case – electricity demand), hourly time series for demand within assessed regions.
• Renewable potential – effective total wind and solar potential of the assessed region, renewable time series estimated for the region based on available data.
• Weather data to compute renewable feed-in timeseries, as well as demand timeseries for e.g. cooling demands.
• Capacities in the base year – includes total installed capacities for production, transmission capacities between neighboring regions for different energy carriers, and storage capacities for different energy carriers and technologies.
• Political targets – targets adopted and maintained by relevant regional and national authorities for decarbonization and energy transition (e.g., targets included in the PPE of Guadeloupe or the LTECV of France).
• Fuel prices and investment costs of technologies.

The output of the model provides an overview of the development of the energy sector within the given timespan, evaluating the needs of the distribution to provide the most beneficial and effective approach. Some of the main outputs of the model consist of:

• Primary energy demand – distribution of the primary energy demand, from different sources and energy carriers.
• Sectoral demand – disaggregated with major sectors – industry, residential, transport, etc.
• Technology investments over time.
• Renewable generation timeseries & energy dispatches and mixes.
• The resulting fossil fuel phase-outs and CO_2_ emission trajectories.

**Figure 2:**
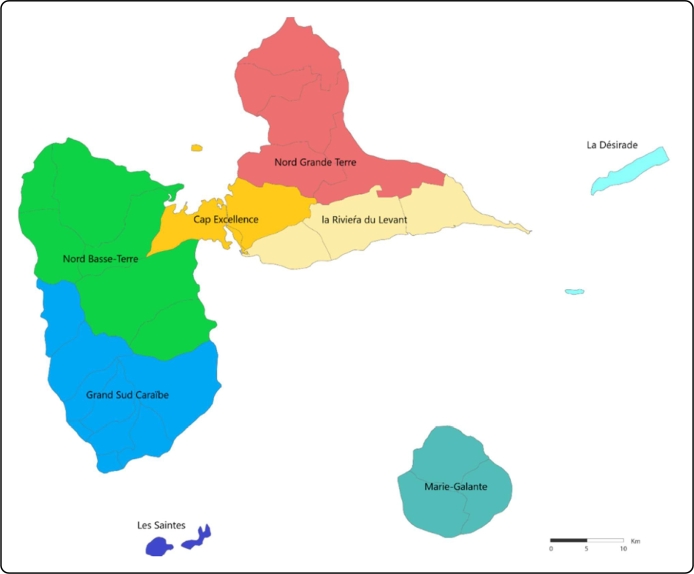
Subdivisions of the Archipelago used for the model.

The work presented builds upon the previous applications of GENeSYS-MOD and implements a model for the integrated energy system of the Guadeloupe archipelago. The group of islands has been divided into eight model regions, as shown in Fig. 2. The modeled timeframe includes the years 2018 to 2050, with 2018 and 2025 being the base years for pre-existing capacities and transmission lines. The model implements an algorithm for the aggregation of the used time series [14] that allows for a flexible amount of time steps within the years, depending on the computational availability of the user. The present model application has been calculated using 120 intra-annual time steps, representing five days at hourly resolution.

A comprehensive overview of the input data, assessed specifically for the case of Guadeloupe is provided in section 3.

### 2.3 Partitioning Guadeloupe into smaller regions

For the desired resolution of the model, the archipelago is divided into eight main regions – based on existing municipal divisions, terrain (different islands within Guadeloupe), and suggestions from local actors (namely EDF Guadeloupe, the major electricity generation utility) for major consumption hotspots.

The Department of Guadeloupe is comprised of two larger administrative aggregates, the Arrondissements of Basse-Terre and Pointe-a-Pitre, which are further divided into 32 administrative communes. The existing administrative structure was used as a basis for dividing the model into regions presented in Fig. 2, as the communes were grouped together to present an accurate subdivision of main islands and provide an outlook of associated physical limitations of transmission and transport.

The eight main regions defined in the model are the following:

• Nord Grande-Terre, La Riviéra du Levant (two regions split from Grande-Terre).
• Nord Basse-Terre, Grand Sud Caraibe (two regions split from Basse-Terre).
• Cap Excellence (an inter-island region for representing the agglomeration around Pointe-a-Pitre).
• La Desirade.
• Marie Galante.
• Les Saintes.

## 3 Input data

Input data for the study was collected based on existing policy documents, data available from open-source databases such as the Opendata portal for EDF Guadeloupe [21], and the French National Institute of Statistics and Economic Studies [22], as well as direct communication with relevant authorities, utility companies, and organizations in Guadeloupe. The entire data set is made available in the supplementary material to the paper [23].

Where data was missing, a set of assumptions and disaggregation practices was used to evaluate and substitute the missing data and define some of the projections utilized in the model for energy potential on islands. The assumptions taken are described further in this section.

### 3.1 Type and installed capacity of power generation resources

Guadeloupe, being an island territory, is heavily dependent on imported resources for energy needs (with a primary energy dependency of greater than 90% in 2021), which is reflected accordingly in the distribution of the electricity sector. To mitigate this, over the past years, the archipelago has seen an increase in the penetration of renewable sources, both through Variable Renewable Energy (VRE) – wind and solar, and others such as hydropower and biomass. A rundown of the main sources of electricity is presented below:

• Total installed capacity of Guadeloupe was 564.3 MW (as of December 2021), the majority of which (329 MW) is supplied by diesel power plants located in the central parts of the archipelago.
• Solar power plants are spread out across the main islands, with the majority of realized potential being located on the island of Grande-Terre. The total capacity of PVs in the archipelago is 87.2 MW.
• The total installed capacity of wind power plants in Guadeloupe is 51.8 MW, which is distributed across 10 onshore power plants – mostly on the northern sections of the archipelago, as well as the island of Marie Galante.
• The archipelago has 14 Run-of-the-river hydropower plants, with a total installed capacity of 10.5 MW. The majority of the power plants are located in the southeastern communes of the island of Basse-Terre.
• Guadeloupe houses the only Geothermal power plant in the Caribbean, the Bouillante power plant, with an installed capacity of 14.5 MW.
• Biogas has limited use in Guadeloupe. Currently, two power plants with a combined installed capacity of 3.7 MW, supply electricity to the archipelago through biogas produced from waste.
• Another important contributor to the electricity sector of Guadeloupe is the Albioma Le Moule power plant, which uses locally produced biomass to produce electricity. The power plant utilizes a byproduct of sugar making – bagasse, to partially substitute coal. The total installed capacity of the power plant is 90.3 MW, 57 MW of which is distributed among bagasse/coal, and 33 MW is solely attributed to coal.
• Only sustainable biomass resources in the form of agricultural and forestry byproducts have been considered for the available bioenergy potentials of the archipelago.

### 3.2 Electricity load demand profile time series

Given the lack of precise time series for the archipelago and its communes, a few assumptions were taken for effectively substituting missing data.

First, for the power sector, the hourly consumption time series of the archipelago has been estimated based on the hourly production time series, which were made openly available by the system operator of Guadeloupe (Électricité de France – EDF). The obtained time series was calibrated based on the annual electricity production and consumption values of the archipelago.

The archipelago was further subdivided into aggregate regions by employing a similar approach – by calibrating regional timeseries with annual communal electricity and total electricity consumption of the islands. This resulted in individual timeseries for 32 communes, with varying demand values and similar profiles.

A substantial difference exists between the energy delivered and the energy billed to customers in Guadeloupe, reaching a total of 205 GWh in 2021. It is noteworthy that due to the inherent limitations of Genesys-MOD in accurately incorporating energy losses, the annual electricity load demand has been approximated as equal to the delivered energy.

### 3.3 Renewable time series

**Figure 3:**
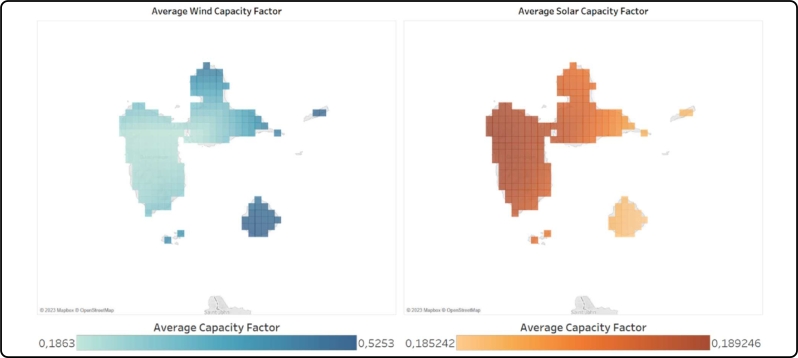
Average yearly capacity factors for solar (right) and onshore wind (left).

The time series for renewable feed-in have been determined using a Python-based tool, built on the Atlite Python package [24]. The script uses ERA5 weather data to provide hourly time series for the chosen weather year for each coordinate, using the information on solar irradiation, wind speeds, and temperature. Fig. 3 shows a map for the generated wind and solar capacity factors.

The coordinates for each region are then clustered into three categories: inferior, average, and optimal. Each category consists of the 33% of the worst, average, and best locations of that specific technology within the region based on the full-load hours across the selected weather year, giving a more granular categorization for the model computation.

### 3.4 Renewable potentials

**Figure 4:**
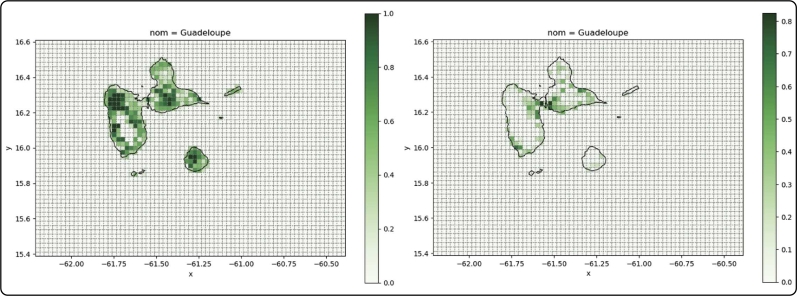
Average surface area per cell for free-standing renewables (left) and rooftop installations (right).

The renewable potentials are calculated using the available surface area, either for utility-scale solar PV or wind installations, using the free available land mass, or for rooftop installations, using information on settlement sizes. Both are computed by combining multiple data sets – the geographical boundaries of the archipelago using a spatial file [25], the surface area information from the Corine Land Cover database [26], and the World Database on Protected Areas (WDPA) [27] to exclude protected areas such as the Guadeloupe National Park on the island of Basse-Terre. The information on the usable area for each grid cell has then been multiplied with a factor for installable capacity per km^2^ for each technology from Agora Energiewende [28], yielding maximum installable capacities for each modeled region (Fig. 4).

### 3.5 Heat demand

Heating demand for Guadeloupe was evaluated for two main categories – electric and non-electric heat demand.

The assessment of the non-electric heating demand in Guadeloupe is based on available annual heating demand data – which was accessible through the annual energy report for four different categories – sugar industry, distilleries, hot water for residential areas, and biogas production [29].

Industrial heating demand was assessed based on consumption from distilleries, sugar refineries, and biogas production. To better assess the demands of Guadeloupe, industrial heating distribution was estimated based on the location of nine main distilleries and two refineries on the islands. Due to a lack of more detailed data, it is assumed that the output of the distilleries and their overall heating demand are roughly the same. For biogas, the heating demand is mainly distributed based on the location of biogas plants (mainly on the island of Basse-Terre).

Disaggregated residential heating data for different regions was estimated based on population figures for different communes and scaled accordingly with the total annual residential heating demand for each of the defined eight regions.

For electric heat demand, the data was sourced based on assumptions of the average consumption of electricity for heating, available through reports conducted on the archipelago for residential, tertiary and industrial sectors [30], [31], [32]. This consumption share was then applied to regional sectoral electricity consumption data to estimate electric heat demand.

### 3.6 Mobility

As an archipelago, Guadeloupe has a naturally complex and diverse system for transporting passengers and various goods throughout its various islands. The system is primarily comprised of three main modes of transportation: Road-based vehicles, maritime transport and aviation (used only as an external transport to and from the archipelago). Road transport is primarily comprised of private vehicles, as well as public interurban transportation, with four main bus lines and ten routes (limited to Basse-Terre and Grande-Terre). Maritime transport has an especially important role in the effective functioning of Guadeloupe, and thus, has an extensive network for interconnecting Grande-Terre and Basse-Terre with the outlying islands. The archipelago has nine main ports spread across all islands, with routes regularly transporting passengers and various goods. Guadeloupe has one major airport, located on the island of Grande-Terre, three km from Pointe-à-Pitre, although it is primarily used for international and regional traffic, and does not service domestic flights to outlying islands. Aside from the above, the archipelago has no public rail transport of significant capacity, aside from privately owned tracks and minor tourist attractions. Due to the lack of precise data, the energy consumption of transport is estimated based on available information – total fuel consumption of the transport sector [29], where available – further disaggregated to separate different modes.

Activity data (for traveled kilometers) were simplified to a single transport category (rather than traditional passenger and freight transport), due to the lack of information relating to freight demands. Data regarding the use of vehicles, as well as distance travelled and average energy consumption per vehicle were obtained based on communication with the representatives of L'ADEME (Agence de la transition écologique), and the model used for their energy demand projections. For the purposes of the model, passenger kilometers used to evaluate specified annual demand were back-calculated based on the total fuel consumption of transport on the archipelago, in combination with other statistical data – vehicle stocks, efficiency, annual travel distances, etc. In line with the above, future fuel demand was assessed by interpolating past values of transport energy consumption (from 2016 onwards, with the exception of 2020–2021), and applying them as a reference for needs of the sector up to 2050.

## 4 Transition pathways and projections

### 4.1 Transition pathways for Guadeloupe's energy landscape

The case study focuses on the development of three different transition pathways with divergent objectives and milestones for accommodating potential energy needs of the archipelago. A brief overview of all energy transition roads is presented below:

1. **Baseline:**
  The **Baseline** case centers on an unconstrained growth scenario, permitting the system to evolve without restrictions concerning emissions or adherence to established national plans for the adoption of renewable energy. Additionally, it accommodates the exploration of fossil fuel technologies, with the potential expansion of coal consumption on the archipelago contingent upon the model's techno-economic assessment. Disregarding CO_2_ emission limits or other political constraints, this scenario represents the pure techno-economic least-cost option for Guadeloupe's energy system transition by 2050.
2. **Independence 2040:**
  This case endeavors to examine an accelerated transition perspective for Guadeloupe, particularly within the critical timeframe of 2035 to 2040. The model is heavily constrained to facilitate the maximum integration of renewable energy sources into the archipelago's energy mix, with a particular emphasis on sectors such as passenger transport and electricity. Notably, this case acknowledges potential challenges, including the prospect of load shedding or inadequate energy supply. In this scenario, Guadeloupe achieves energy independence and self-sufficiency by the year 2040.
3. **Independence 2050:**
  This scenario adopts a relatively pragmatic and grounded strategy to attain energy independence in the archipelago, with less ambition in the near-term future than the *Independence 2040* scenario. It exhibits a greater openness to maintaining traditional technologies in Guadeloupe, allowing them a longer operational lifespan to meet the ongoing energy demand. The approach is characterized by a more gradual transition, implementing pivotal shifts toward renewable energy sources over a more prolonged timeframe. In this transition pathway, Guadeloupe achieves energy independence and self-sufficiency by the year 2050.

### 4.2 Projecting load demand

The provisional plan of the electricity sector of Guadeloupe by EDF [33] serves as the foundation for projecting the final energy demand of various sectors within all storylines. It delineates two distinct pathways – Azure and Emerald – projecting annual load demand until 2038 based on two key characteristics: population and macro-economy. This study focuses on the Azure scenario, extending the analysis to 2050. In this scenario, the demographic decline and actions to control energy demand continue to push the consumption trajectories downward. However, energy consumption decreases in the short term, but increases in the medium and long term, which is due to the integration of electric vehicles in Guadalupe.

Load demand projections used for the elaboration of the model are primarily sourced from two available development plans, the Programmation Pluriannuelle de l'Énergie (PPE) and the Bilan de l 'énergie (Energy Report), which ranges from 2018 to 2021.

Initial projections for the archipelago include an annual estimation of the electricity demand for 2023, 2028 and 2033, as well as legacy data for 2018 and 2021. The reference values for past years are also further subdivided by territorial aggregates defined in the scope of the study, as well as main consumption sectors.

Projections for the evolution of demand in Guadeloupe were estimated in 5-year intervals, in the span of 2025–2050. Data used in the scope of the assessment is partially based on the model developed by EDF to forecast the development of electricity and mobility sectors in the archipelago [33]. In the electricity sector, we applied a multiple linear regression model with two independent variables – specifically, population and GDP – and one dependent variable, the electricity load demand. This methodology was employed to broaden the scope of the Azure scenario projection for electricity demand, extending it to the year 2050.

In the mobility sector, drawing from insights in the same report, we utilized interpolation techniques to project the anticipated reduction in passenger kilometers up to the year 2050. Regarding the heat sector, the portion supplied by electricity was estimated based on the projected electricity load demand. Meanwhile, the non-electric heat demand was assumed to remain constant throughout the study period.

### 4.3 CO_2_ emission reduction

**Figure 5:**
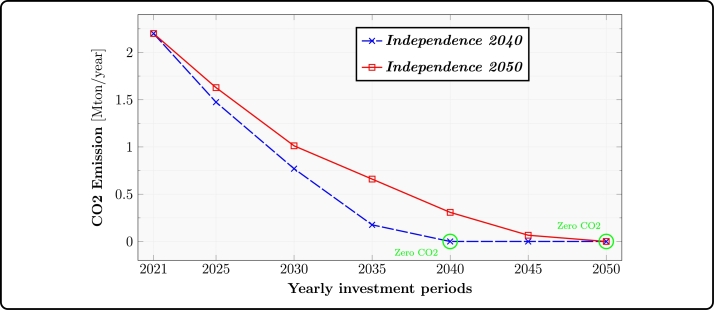
Emission reduction trends along the transition roads of *Independence 2040* and *Independence 2050*.

While defining the scenarios, specific upper limits for CO_2_ emissions were established, illustrating a gradual reduction over the temporal span of the study. Fig. 5 visually represents the established emission thresholds for both the *Independence 2040* and *Independence 2050* transition pathways. Noteworthy is the fact that *Independence 2040* requires a more pronounced decline in CO_2_ emissions, with the objective of achieving a decarbonized energy system a decade earlier than the *Independence 2050* trajectory.

## 5 Model results

### 5.1 Power sector

The power sector of Guadeloupe experiences significant shifts in the structure of energy supply, with the scenario settings having a profound impact on the type and magnitude of adopted new technologies.

**Figure 6:**
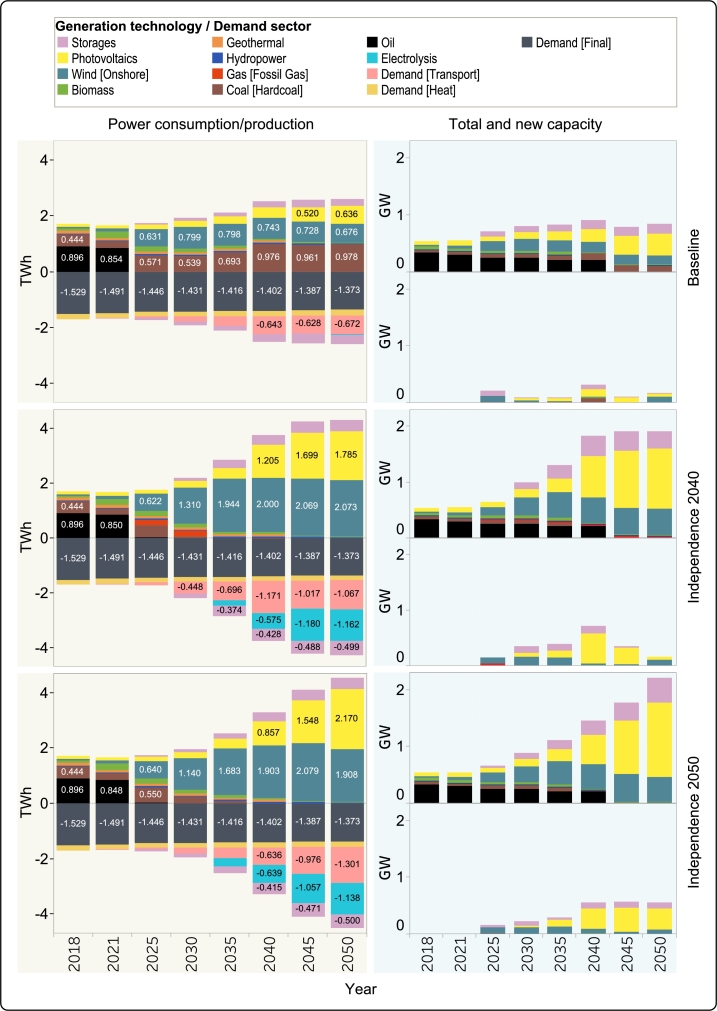
Left: Comparative analysis of annual electricity production and consumption across all scenarios. Right: Overview of total (top) and newly installed (bottom) capacity for diverse generation resources in the scenarios.

As can be seen in Fig. 6, the electricity demand gradually increases from 1.7 TWh to around 2.4 TWh from 2040 onward in the *Baseline* scenario. Due to the lack of any emission constraints or quotas for renewable energy adoption, the electricity sector still has a significant share of coal power plants in the system, increasing even further over the years to satisfy growing demand, with minor added capacity in 2025 and 2035, and a significant addition in 2040 of 81.6 MW. Nevertheless, the overall supply has a higher renewable share, as the oil power plants operating in Cap Excellence during reference years are decommissioned swiftly by the model.

The primary sources of renewable energy in the electricity system are wind and solar, with roughly equal shares in electricity generation, but more installed capacity for solar due to the lower full-load hours. The installed capacity for wind power plants is added most significantly in 2025 and 2050 with around 100 MW, but the power production remains within the range of 0.6–0.8 TWh throughout the entire modelling period. Solar PV plants are adopted in the system gradually, with new capacity added in each of the modeled years, with generation, peaking in 2050 with 0.64 TWh. Other sources of renewable energy already present on the island for electricity generation, especially biomass, are phased out. This is because the model deems the available biomass to be more valuable in other end-use sectors rather than electricity generation.

The *Independence 2040* and *Independence 2050* scenarios provide a drastically different outlook for the development of the electricity sector, as the emergence of electric transport and hydrogen production drive up the demand in the future.

In the transition plan outlined in *Independence 2040*, there is a critical need to significantly reduce CO_2_ emissions by 2030. However, there is a constraint on investments in each generation resource within each investment period, regarding the feasibility of implementing the plan within a short timeframe. As depicted in Fig. 6, as an optimal solution and in recognition of the constraints of the shorter transition period, an additional intermediate technology in the form of gas is introduced in 2025 and 2030 to entirely eliminate coal and oil, which have higher emission production. It is worth noting that a looser constraint on the periodic investment limit of different technologies will lead to increased investment in wind power plants, while a tighter constraint may prompt investment in other new technologies. In this scenario, by 2030, the electricity supply is dominated by renewable sources – wind (with 1.310 TWh), solar (0.245 TWh), and existing other sources (Hydropower, geothermal and biomass). Post-2030, the adoption of variable renewables (VRE) experiences significant growth, with wind and solar exceeding 2 TWh and 1.25 TWh in 2040, respectively. Total installed capacity (generation and storage) reaches 1.83 GW in the same period and further grows to 1.91 GW in 2045 and 2050.

Contrary to the *Independence 2040* scenario, *Independence 2050* (see Fig. 6) experiences a more gradual transition. Consequently, some fossil fuel based generation persists until 2035. Similar to the *Independence 2040* scenario, the electricity supply is heavily dominated by wind and solar in the future years. However, there is a shift in the generation mix, with solar PVs exceeding wind in 2050 (2.2 TWh and 1.9 TWh for solar and wind respectively). In this transitional pathway, post-2035 allowances for CO_2_ emissions are allocated to the transportation sector, contributing to an early decarbonization of the power sector.

As a final point, it should be noted that in every scenario, the development of further VRE capacity necessitates the implementation of additional storage technologies, mostly in the form of batteries. Geothermal power generation, which could provide some baseload electricity, is not expanded beyond the currently installed capacities, even though it exists as a technology option in the model. This shows that from an economic perspective, the model sees VRE + storages as a cheaper solution compared to further geothermal installations. However, this finding could also partially be based on a possible underestimation of the model regarding flexibility requirements (see Section 6).

### 5.2 Transportation sector

**Figure 7:**
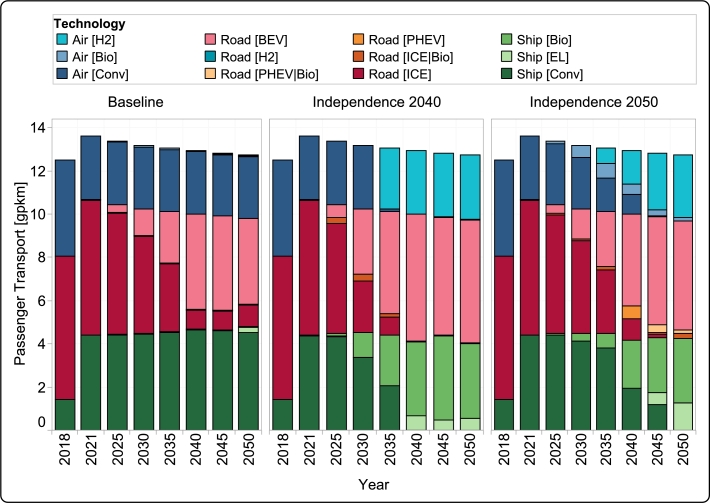
Passenger transport output attributed to diverse technologies across all scenarios spanning the years 2018 to 2050.

The transport sector exhibits significant differences in the three scenarios (see Fig. 7). Reference years (2018 and 2021) are nearly completely dominated by conventional fossil fuel-based transport, with a nearly negligible share of electric vehicles in 2021. In addition, the transport sector is the last sector to be is decarbonized in *Independence 2040* and *Independence 2050*.

In the *Baseline* scenario, road passenger transport transitions largely to electric vehicles, with their share gradually increasing from 2025. However, conventional vehicles still persist in the mix even in 2050. Other modes of transport have a largely stagnant trend, although some electric ships appear as a minor alternative to conventional transport by 2050. It is worth noting that there are already plans to increase the integration of electric vehicles in Guadeloupe. In this regard, we have factored in a minimum share of electric vehicles in the model. The shift to electric vehicles is influenced, in part, by the minimum limit we have considered for electric vehicles in all scenarios. In the *Independence 2040* scenario, the transition towards renewable energy extends to all major modes of transport:

• From 2035 onward, Air transport is nearly completely devoid of conventional fuels and relies exclusively on hydrogen for energy needs.
• From 2025 onward, conventional fuel is gradually replaced with biofuel and electric ships. Renewable sources fully overtake fossil fuels in ships by 2040.
• For road transport, the transition towards electric vehicles happens more swiftly. Conventional vehicles are completely phased out by 2040, in favor of battery electric vehicles. The model also introduces plug-in hybrid electric vehicles as a transitional technology between 2025–2035.

The *Independence 2050* scenario largely follows the same trend, albeit with a more gradual transitional timeframe, all major modes of transport are affected (see Fig. 7):

• Air transport introduces hydrogen after 2030. Conventional fuels are gradually replaced with a mix of hydrogen and biofuel, and are fully substituted in 2050.
• Conventional, fossil-fueled ships remain in the transport mix until 2045, and are only fully replaced by 2050. It should be noted that the share of electric ships in *Independence 2050* is higher than *Independence 2040* for 2045 and 2050.
• Replacement of conventional road vehicles is more gradual, the penetration of battery electric vehicles continues to grow steadily from 2025, alongside the introduction of a small share of hybrid vehicles. Conventional vehicles are completely eliminated by 2050, and are mostly dominated by BEVs, with a minimal share of hybrids.

### 5.3 Heat sector

The heat sector is assessed separately for residential/commercial and industrial segments. In both sectors, the total heat demand experiences a marginal reduction until 2050, following the current projections.

**Figure 8:**
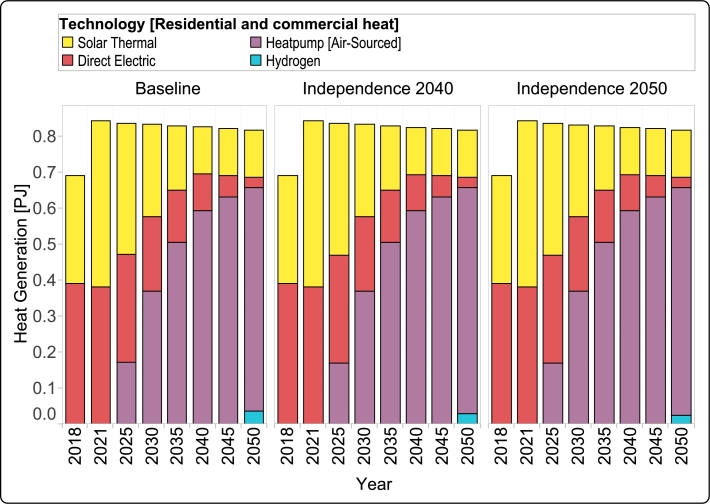
Contribution of different technologies to the annual production of residential and commercial heat for all scenarios from 2018 to 2050.

For the residential and commercial sectors, solar thermal and direct electricity were the primary sources of heat production in the base years (2018 and 2021), as shown in Fig. 8. The plan in Guadeloupe is to expand the use of solar thermal and replace direct electricity with this technology. However, the output of the GENeSYS-MOD indicates that solar PV, coupled with heat pumps may be a more effective solution. To achieve this transition, increased investment in the power sector is required to support heat pump deployment. Despite the necessity for increased investment in the power sector, the notable efficiency of heat pumps leads to a gradual rise in their adoption to meet the heat demands of residential and commercial sectors. An intriguing observation lies in the consistent trend of escalating heat pump deployment across all scenarios. This pattern affirms that the choice to adopt heat pumps is purely economic and is not contingent on the constraint of reducing CO_2_ emissions. However, it is worth noting that while heat pumps account for approximately 75% of residential/industrial heat generation, in all three scenarios, the model still incorporates solar thermal as a complementary technology to meet the overall residential/commercial heat demand in 2050. Additionally, the results also indicate some very minor emergence of hydrogen usage for heat production in these sectors during the final investment period.

**Figure 9:**
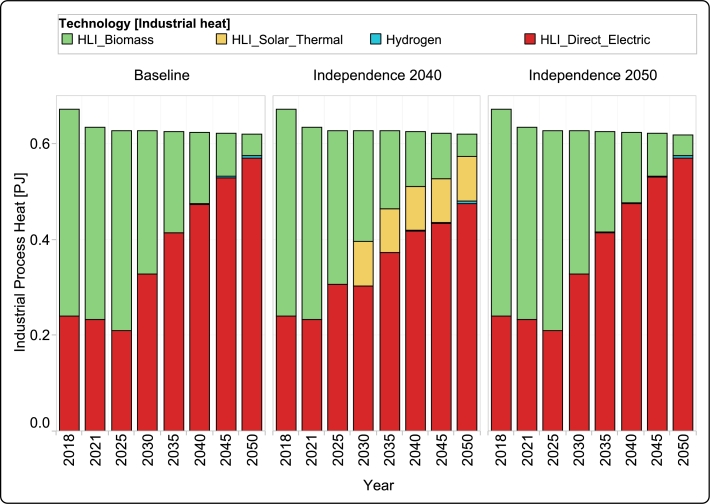
Contribution of different technologies to the annual production of industrial heat for all scenarios from 2018 to 2050.

Fig. 9 illustrates the technologies employed for industrial heat production in all three scenarios. In the base years (2018 and 2021), biomass and direct electricity were the primary contributors to the industrial heat production. As shown in the figure, both the *Baseline* and *Independence 2050* scenarios exhibit a similar trend. By 2025, the use of biomass for industrial heat production sees a minor increase, but this trend reverses after 2025, resulting in more than 90% of industrial heat being generated through electricity.

A comparison between these two scenarios reveals that the decision to shift industrial heat production to direct electricity is purely economic, given the absence of CO_2_ emission constraints in the *Baseline* scenario and the underutilization of biomass capacity. However, the divergence in the *Independence 2040* scenario from the other two is attributed to the biomass limit imposed in the model, capped at three petajoules. In *Independence 2040*, driven by the imperative to significantly reduce CO_2_ emissions, the model allocates biomass to the transportation sector, leading to an investment in solar thermal for industrial heat production. Nevertheless, in this scenario, the contribution of direct electricity to industrial heat production increases steadily toward 2050, following a similar trend as the other two scenarios.

### 5.4 Primary energy

**Figure 10:**
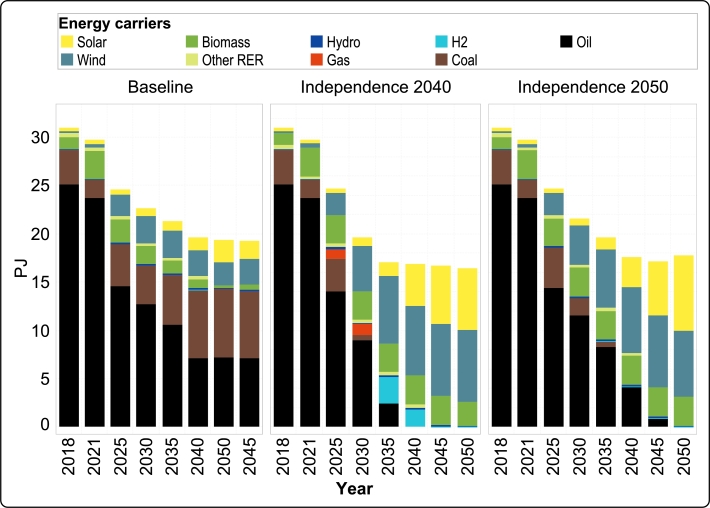
Guadeloupe's annual primary energy for all scenarios from 2018 to 2050.

Primary energy in Guadeloupe is characterized by a complete dominance of fossil fuels in the reference periods (2018 and 2021), with only a minor share of renewable energy in the energy mix, as shown in Fig. 10. As a purely economic decision, this largely persists in the *Baseline*, even though the total primary energy consumption decreases significantly due to shifts in transport and electricity generation and relevant improvements in efficiency. The primary sources of energy are still oil – primarily used for transport, as the electricity generation from diesel plants is mostly halted in 2025, coal – with a larger share due to increased electricity generation, as well as wind and solar, which replace biomass as the major renewable sources of the archipelago.

In *Independence 2040*, this condition changes drastically as the share of fossil fuels starts to decrease rapidly to accommodate the early transition goal (i.e., reduction in CO_2_ emission). Oil as a source for transport persists through 2035; however coal is eliminated in 2030 as the demand on coal fired power plants is reduced due to the early transition of the power sector. From 2040 onward, the energy mix is comprised of hydrogen (which is introduced as a substitute for conventional fuel since 2035), biomass, wind and solar.

As evident in the figure, *Independence 2050* follows the same trend as *Independence 2040*, grounded in similar reasons but with a delay due to less restrictive CO_2_ reduction constraints. Fossil fuels remain in the energy mix up to 2045, the share of biomass largely remains stagnant from 2025 onward, and there is a steady increase in the penetration of solar and wind. A visible difference between *Independence 2040* and *Independence 2050* is the investment in gas-based power plants in the first transition road. The primary reason for this investment is the growth rate limit imposed on various technologies considered in GENeSYS-MOD. This constraint is necessary to prevent a large investment in a specific technology within a specific period, which may not be practically implementable. In *Independence 2040*, with the imperative to significantly reduce CO_2_ emissions by 2030 and decrease coal usage, the model invests in gas-based power plants due to the mentioned growth limit for wind. Removing this constraint or increasing the growth rate factor would eliminate the investment in gas-based power plants.

It is worth noting that the availability of biomass has been limited to three petajoules in all transition roads. According to the results, both *Independence 2040* and *Independence 2050* maximally utilized this resource. This implies that the higher availability of biomass significantly impacts the energy transition road. Therefore, investment in biomass production is essential for achieving energy independence in the Guadeloupe energy system.

### 5.5 Emissions

**Figure 11:**
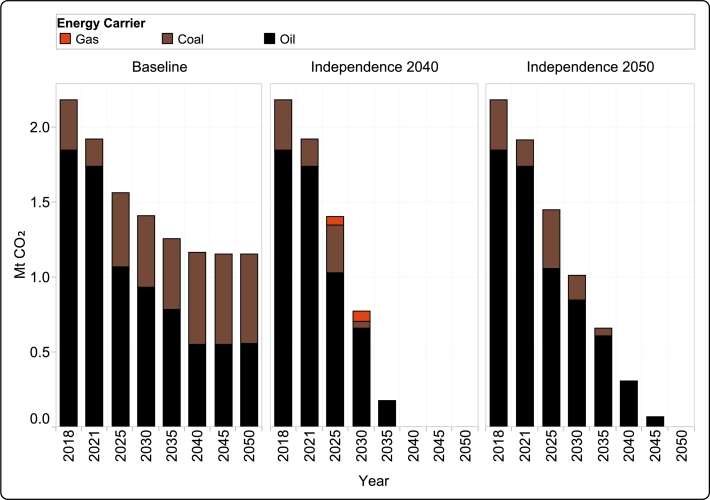
Annual CO_2_ emissions across all transition roads from 2018 to 2050.

Emissions in Guadeloupe are from two main sources: oil and coal. Reference years (2018 and 2021), already exhibit a decreasing trend, as indicated by the input data, which continues in all transition roads, as shown in Fig. 11.

For *Baseline*, this trend is most noticeable in the early years, as total emissions are nearly halved from 2018 to 2040. This change can largely be attributed to the overall decrease in energy consumption and penetration of more renewable energy sources in the energy mix and electric vehicles in the transport sector. However, as the model increasingly utilizes coal for electricity production, the emission decrease begins to stop in 2040, with only a negligible change in the later decade. Furthermore, as seen in Fig. 11, the increased reliance on coal results in a prominent shift within the emission structure, and more than 50% of all emissions are derived from coal from 2040 onward, as opposed to 15% in 2018. The cumulative CO_2_ emissions in the selected years from 2025 onward is 7.66 Mton.

The emission reduction in *Independence 2040* and *Independence 2050* stems from the stringent constraint imposed on the allowed CO_2_ emissions during each investment period and scenario, as depicted in Fig. 5. In both transition roads CO_2_ emissions approach their maximum limit, with the exception of the year 2025.

In *Independence 2040*, emissions decrease rapidly in the span of 2018–2035. It is noteworthy, that due to the introduction of gas as a transitional technology in the electricity generation mix in 2025, some emissions from gas are present in 2025 and 2030 (see Fig. 11). Minor emissions from oil still remain through 2035 due to a small share of conventional road vehicles in the transport pool. However, as the sector completely transitions in 2040, the corresponding emissions are eliminated. The cumulative CO_2_ emissions in the selected years from 2025 onward is 2.35 Mton in this transition road.

The emission reduction in *Independence 2050* is still considerably larger than in the *Baseline*; however the timeline for achieving net zero emissions is shifted to 2050. As the model did not invest in gas-fired power plants, the emission sources are comprised of only oil and coal. As notable in Fig. 11, total emissions from coal increase in 2025 as electricity generation shifts from oil to coal; however with the introduction of more solar and wind, it starts to steadily decrease afterwards. Only transport-based emissions remain in 2040 and 2045, most from conventional fuel ships, as well as some minor emissions from road transport. As the sector completely transitions to renewable sources, the energy sector is completely decarbonized in 2050. The cumulative CO_2_ emissions from the selected years starting in 2025 amount to 3.54 Mton, which is much higher than in the *Independence 2040* scenario but significantly less than in the *Baseline* transition road.

### 5.6 Short-term dispatch of the electricity sector

**Figure 12:**
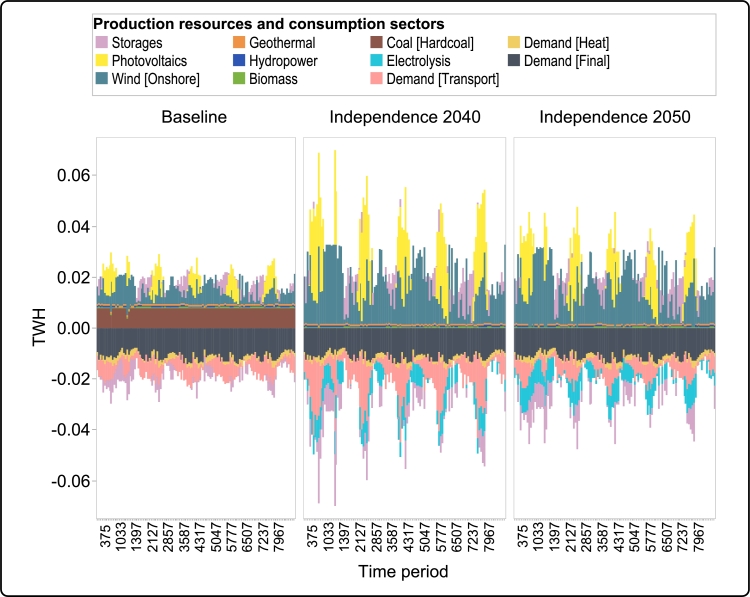
Comparison of short-term dispatch structures for different transition roads during 2040.

The variation between different transition roads has a profound effect on the short-term dispatch structure throughout the time period. Fig. 12 shows the short-term operation of Guadeloupe's energy system for the three transition roads in 2040.

As can be seen in this figure, the dispatch structure in the *Baseline* is largely consistent throughout, as the base demand is mostly satisfied with coal-fired power plants, and input from biomass and hydropower does not shift significantly. The variable production of wind and solar is compensated with storage technologies.

The dispatch structure is drastically different in the *Independence 2040* transition road. As noted in Fig. 12, since the system relies only on renewable sources for electricity production, there is a greater need for additional storage technologies, which is satisfied through investments in conventional battery storage and electrolysis for hydrogen production.

The dispatch structure is similar for the *Independence 2050* transition road as well, although with some differences pertaining to the adoption of new technologies – particularly the differences in the electrification of road and ship transport. While the electricity sector in both *Independence 2040* and *Independence 2050* transition roads is fully decarbonized by 2040, *Independence 2040* necessitates significantly higher investment in renewables – specifically solar PV – to provide sufficient energy for the earlier electrification of the transportation sector. This requirement is reflected in the yearly dispatch patterns shown in Fig. 12.

## 6 Limitations

While our model and its application are able to give interesting insights into development strategies for the energy system and its different subsectors for the Guadeloupe archipelago, as with all numeric models, it is only a stylized representation of the real world and therefore comes with limitations that need to be acknowledged. Being a linear model, GENeSYS-MOD simplifies investment decisions by making them continuous, which can distort and underestimate upfront investment costs into infrastructure (e.g. for BEV charging infrastructure). Additionally, being a cost-optimizing model, it heavily relies on the used input data for technology and fuel costs. This, coupled with the linear model setup, can favor corner solutions, where the model will fully maximize the usage of one single technology up to the absolute maximum, if it deems it the cheapest option, even when in real life, one would expect more heterogeneity. These issues are sought to be limited in their weight by the way that the model data is set up: for renewable energy sources, for example, each technology is split into several categories based on their generation potential. This leads to a more heterogeneous distribution of levelized costs of electricity generation (LCOE) which in turn produces more realistic results by giving this piece-wise linear cost curve [34].

Also, the large nature of the model set-up in terms of technology variety and sectoral coverage means that in order to keep computation within a reasonable time-frame, a time-series reduction algorithm [14], [35] has been applied. This means that not all 8760 hours of the year are considered, but are aggregated into 120 intra-annual time-steps. This means that flexibility requirements could be slightly underestimated, which could also explain the choice of the model not to expand geothermal electricity generation and instead invest so heavily into wind and solar power.

## 7 Concluding remarks

This study utilized the Global Energy System Model (GENeSYS-MOD) to explore the intricate dynamics of Guadeloupe's energy transition, spanning power, transportation, heat, and primary energy sectors. Two transition scenarios aimed at achieving energy independence by 2040 and 2050 were compared against a *Baseline* scenario lacking explicit energy independence goals. The findings revealed nuanced trends in the energy landscape, offering valuable insights for Guadeloupe's sustainable development.

In the power sector, the *Baseline* scenario illustrated a gradual transition toward renewable energy, while still maintaining a significant dependence on coal. In contrast, the *Independence 2040* and *Independence 2050* scenarios pursued more assertive transitions, driven by CO_2_ emission reduction goals. Notably, the power sector decarbonized ahead of the transport sector in both *Independence 2040* and *Independence 2050*. This is especially relevant in the wake of sector-coupling, as without renewable – and thus emission-free – electricity, the emission reduction would not be nearly as significant.

However, the transportation sector generally underwent a transformative evolution, with a rise in electric vehicles (for road-based transport), hydrogen, and biofuels (for shipping and aviation). With the stricter emission targets, *Independence 2040* showcased a rapid transformation in a short timeframe, whereas *Independence 2050* exhibited a more gradual evolution, emphasizing diverse solutions across major transport modes.

For residential/commercial heating, the findings suggested a transition towards heat pumps driven by their efficiency, overshadowing the prevalence of solar thermal and direct electric heating. Concurrently, industrial heat production uniformly transitioned from biomass to direct electricity in all transition scenarios. Remarkably, the heat sector, encompassing both residential/commercial and industrial aspects, demonstrated the least susceptibility to the constraints imposed in *Independence 2040* and *Independence 2050*.

While the study provides a comprehensive exploration, certain aspects merit attention in future research. The cooking sector, including its technology options and energy demand, should be taken into account. Also, the employed limit of three petajoules on the availability of sustainable biomass is an assumption due to the lack of data, and this assumption has implications for the results. A more precise input for this limit would contribute to a more solid and reliable output.

Overall, the current government targets are not ambitious enough given global warming and the need to reduce emissions. However, the transformation is technically feasible and enables full energy independence for the archipelago by 2040. Future government plans should reflect these possibilities and ramp up their ambition regarding decarbonization options, starting with an expansion of RES in the electricity sector.

## Data and Scripts

The input data and scripts used in this study are publicly available in version v2025-01-16 of the GENeSYS-MOD-Guadeloupe repository on GitHub [23].

## Funding

This work was supported by the 10.13039/501100000780European Commission under the Horizon 2020 project “TransformAr” (grant number 101036683).

## CRediT authorship contribution statement

**Mostafa Barani:** Writing – review & editing, Writing – original draft, Visualization, Validation, Supervision, Software, Resources, Methodology, Investigation, Formal analysis, Data curation, Conceptualization. **Konstantin Löffler:** Writing – review & editing, Writing – original draft, Visualization, Validation, Supervision, Software, Project administration, Methodology, Investigation, Formal analysis, Data curation, Conceptualization. **Luka Garibashvili:** Writing – original draft, Visualization, Validation, Software, Investigation, Formal analysis, Data curation. **Pedro Crespo del Granado:** Validation, Supervision, Resources, Project administration, Funding acquisition, Conceptualization.

## Declaration of Competing Interest

The authors declare that they have no known competing financial interests or personal relationships that could have appeared to influence the work reported in this paper.
